# Process evaluation of a community advisory board for a multi‐site clinical trial addressing dementia literacy and linkage to dementia care

**DOI:** 10.1002/alz.092928

**Published:** 2025-01-09

**Authors:** Hae‐Ra Han

**Affiliations:** ^1^ Johns Hopkins University School of Nursing, Baltimore, MD USA

## Abstract

**Background:**

Community engagement is a key strategy to promoting health equity. While community advisory boards (CABs) are used as a popular tool for community engagement, the process of forming CABs and maintaining them is mostly missing in the literature. The purpose of this study is to showcase a set of processes our collaborative team went through to form and implement a CAB in the context of dementia literacy and linkage to dementia care research.

**Method:**

PLAN—Preparing for healthy aging through dementia Literacy education And Navigation—is an ongoing, multi‐site clinical trial involving community‐dwelling Korean American older adults (65+ years) with probable dementia and their care partners in the US. Data for the current analysis came from multiple sources: monthly CAB meeting minutes, study team meeting minutes, and notes taken during individual discussions with CAB members. We used content analysis to identify common themes across these data sources.

**Result:**

We identified three main stages in relation to CAB: Formation, Implementation, and Sustainability. Formation stage was mainly to build genuine connections as a team while clarifying the research purpose and establishing the mission and goals of the CAB. Key activities for the Implementation stage included: Strategizing for community outreach to build trust with the local community; advocating for the rights of vulnerable research participants such as those with limited English proficiency and/or with probable dementia with/without care partners; ensuring that the language of research materials and message is culturally relevant, friendly, and easy to understand; increasing awareness and building bridges between science, patients, caregivers, and families; strategizing how to make clinical research accessible to everyone; and creating a CAB‐led community engagement project to share experiences and perspectives. In the current, Sustainability stage, we are actively evaluating our partnership processes, while determining next steps to scale and adapt the PLAN program.

**Conclusion:**

When implemented effectively, CABs can help center the perspectives and experiences of marginalized groups such as those with limited English proficiency. It would be important for future research to evaluate in greater depth what components make a CAB partnership successful so that more resources related to CAB formation, implementation, and sustainability can be utilized for research.

